# Drug-Associated Gastropathy: Diagnostic Criteria

**DOI:** 10.3390/diagnostics13132220

**Published:** 2023-06-29

**Authors:** Dmitry S. Bordin, Maria A. Livzan, Olga V. Gaus, Sergei I. Mozgovoi, Angel Lanas

**Affiliations:** 1 A.S. Loginov Moscow Clinical Scientific Center, Department of Pancreatic, Biliary and Upper Digestive Tract Disorders, 111123 Moscow, Russia; 2 Department of Propaedeutic of Internal Diseases and Gastroenterology, A.I. Yevdokimov Moscow State University of Medicine and Dentistry, 127473 Moscow, Russia; 3 Department of Outpatient Therapy and Family Medicine, Tver State Medical University, 170100 Tver, Russia; 4 Department of Faculty Therapy and Gastroenterology, Omsk Sate Medical University, 644099 Omsk, Russia; 5 Department of Pathological Anatomy, Omsk Sate Medical University, 644099 Omsk, Russia; 6 Digestive Diseases Service, Aragón Health Research Institute (IIS Aragón), University Clinic Hospital, University of Zaragoza, 50009 Zaragoza, Spain

**Keywords:** gastritis, drug-induced gastric damage, NSAIDs, aspirin, anticoagulants, mycophenolate, checkpoint inhibitors, iron therapy, glucocorticoids

## Abstract

Drugs are widely used to treat different diseases in modern medicine, but they are often associated with adverse events. Those located in the gastrointestinal tract are common and often mild, but they can be serious or life-threatening and determine the continuation of treatment. The stomach is often affected not only by drugs taken orally but also by those administered parenterally. Here, we review the mechanisms of damage, risk factors and specific endoscopic, histopathological and clinical features of those drugs more often involved in gastric damage, namely NSAIDs, aspirin, anticoagulants, glucocorticosteroids, anticancer drugs, oral iron preparations and proton pump inhibitors. NSAID- and aspirin-associated forms of gastric damage are widely studied and have specific features, although they are often hidden by the coexistence of *Helicobacter pylori* infection. However, the damaging effect of anticoagulants and corticosteroids or oral iron therapy on the gastric mucosa is controversial. At the same time, the increased use of new antineoplastic drugs, such as checkpoint inhibitors, has opened up a new area of gastrointestinal damage that will be seen more frequently in the near future. We conclude that there is a need to expand and understand drug-induced gastrointestinal damage to prevent and recognize drug-associated gastropathy in a timely manner.

## 1. Introduction

Drug therapy is one of the key elements of the armamentarium employed to treat many different diseases. Clinical trials and real changes in the mucous membrane are associated with the actions of various etiological factors, while drug-induced gastritis is characterized by various structural changes in the gastric mucosa with minimal signs of inflammation, which has led to the more frequent use of the collective term “gastropathy” as a synonym for drug-induced gastritis. In this case, damage to the gastric mucosa can be acute or chronic.

This publication was prepared in order to systematize the available data on modern diagnostic criteria for drug-induced gastropathy (DIG)—lesions of the gastric mucosa associated with a negative manifestation of either a drug or its metabolites.

The diagnosis of DIG should be based on the identification of gastric damage chronologically caused by the use of the drugs followed by recovery or a pronounced decrease in the signs of gastropathy after discontinuation of the medication. In this regard, thorough history taking, including medical history, with a clarification of previous signs is one of the first reasons for a diagnostic search in the case of DIG [1].

Clinical symptoms of DIG are variable. Often, patients with DIG may be asymptomatic or have mild symptoms which can be overshadowed by the presence of symptoms of the underlying disease, which means that it is difficult to assess or detect the presence of DIG in a timely manner. Some patients, however, may have dyspepsia, as well as clinical symptoms showing that other parts of the digestive tract are concerned. Finally, patients with DIG may have more severe symptoms, including severe pain or a complicated course with anemia, or overt bleeding and perforation associated with ulceration.

The clinical signs of DIG upon physical examination are not specific and often reveal only signs of the underlying disease treated with the drug inducing the gastroduodenal damage. In some cases, epigastric tenderness can be present, and in cases of a complicated course, pallor of the skin and the presence of visible mucous membranes, hypotension and compensatory tachycardia are common.

Non-invasive tests have low informative value but may have diagnostic value, either for detecting a complicated course of the disease (signs of posthemorrhagic iron deficiency anemia according to the results of a blood test, a positive fecal occult blood test, transferrin or fecal hemoglobin) or when conducting a differential diagnosis with other types of gastritis (for example, a gastropanel with the determination of parietal cell antibodies in autoimmune gastritis).

The next step in the diagnostic process of DIG is an endoscopic examination with the collection of gastric biopsy specimens. It should be taken into account that the majority of endoscopic and histopathological changes in the gastric mucosa caused by taking drugs are nonspecific and reflect inflammation, erosions or uncomplicated or complicated ulcers. Biopsies of the gastric mucosa, however, may show morphological changes in the so-called reactive gastropathy pattern, most clearly represented as damage induced by non-steroidal anti-inflammatory drugs (see below). However, the coexistence of H. pylori-associated gastritis often hides these features linked to DIG. Nevertheless, there are individual characteristic features of the morphological picture associated with the action of a particular drug agent, which may be specific. These features are reviewed in this article.

## 2. Non-Steroid Anti-Inflammatory Drugs/Acetylsalicylic Acid (NSAIDs/Aspirin)

The first description of the endoscopic picture of the damage to the gastric mucosa associated with the use of aspirin was published by A. Douthwait and J. Lintoff in 1938 [2].

### 2.1. Epidemiology

NSAIDs are one of the most commonly prescribed classes of medication with a wide range of indications and availability in over-the-counter forms. According to some studies, the prevalence of NSAID and aspirin use among older people is 24.7% [3]. Gastric erosions occur in approximately half of patients receiving NSAIDs, and peptic ulcer disease occurs in 15–30% of cases. Symptomatic peptic ulcers can be observed in 3–4.5% of patients taking NSAIDs, and serious complications (perforation, obstruction or bleeding) occur in approximately 1.5% of patients after 1 year of treatment [4].

According to two large cohort studies, ESTHER (N = 7737) and British Biobank (N = 213,598), taking low doses of aspirin is an independent risk factor for the development of gastric and duodenal ulcers in the early period after the start of treatment [5]. The stomach and duodenal ulcer risk ratios were found to be 1.82 [1.58–2.11] and 1.66 [1.36–2.04] in the case of British Biobank and 2.83 [1.40–5.71] and 3.89 [1.46–10.42] in the ESTHER study, respectively.

According to data from Spain, the mortality rate associated with the use of NSAIDs or aspirin is 5.6%, which is equivalent to 15.3 cases of death per 100,000 users [6].

### 2.2. Risk Factors

Risk factors for the development of NSAID/aspirin-associated gastropathy include >60-year-olds (and, in particular, >70-year-olds), high-dose NSAID treatment, a previous history of peptic ulcers with or without complications, co-therapy with low-dose aspirin, anticoagulants, serotonin re-uptake inhibitors or steroids and *H. pylori* infection [7,8,9].

### 2.3. Mechanism of Gastric Damage

Gastrointestinal-associated NSAID/aspirin damage is based on the blockade of the enzyme cyclooxygenase (COX), which regulates the synthesis of prostaglandins from arachidonic acid. COX exists in two isoforms: structural COX-1 and induced COX-2. The COX-2 isoform is not detected in normal tissues. Its expression is induced by inflammatory mediators (lipopolysaccharides, interleukin-1, tumor necrosis factor alpha, macrophages, monocytes) and causes all the clinical manifestations of inflammatory processes: soreness, fever, swelling and dysfunction. Therefore, it is the blockade of COX-2 that causes the main targeted pharmacological effects of NSAIDs/aspirin, including the anti-inflammatory, analgesic and antipyretic. At the same time, COX-1 blockade induces a systemic decrease in the synthesis of prostaglandins (PGs), which have cytoprotective effects.

It has been established that PGE2 inhibits the formation of H+ ions and pepsinogen in the stomach, reducing the volume of gastric secretion and its acid and peptic activity; however, the main effect is the increase in the production of mucus and bicarbonates, stimulation of the processes of cell proliferation and physiological regeneration of the epitheliocytes of the gastric mucosa [10]. Thus, a decrease in PG synthesis is associated with a decrease in the resistance of the gastric mucosa [11], as well as a reduction in the gastric mucosal blood flow due to the ability of NSAIDs/aspirin to inhibit the synthesis of nitric oxide (NO) through the suppression of the activity of the NO synthetase enzyme [12]. At the same time, a decrease in the formation of PG leads to the activation of the lipoxygenase pathway, with an increase in the synthesis of leukotrienes (LTs), primarily LT-B4, and pro-inflammatory cytokines (C5-compliment, tumor necrosis factor-α), which aggravate the inflammation and ischemia of the gastric mucosa [13,14].

The direct (topical) interaction between NSAIDs and phospholipids and the uncoupling of oxidative phosphorylation in mitochondria cause cell membrane damage, with a disruption of the phospholipid layer and tight junctions. This action increases transcellular permeability. The inhibition of COX, as a systemic effect, reduces microvascular blood flow, and luminal aggressive factors modify and amplify this reaction, leading to inflammation, erosions and ulcers [9].

Depending on their blockade of one COX isoform or another, NSAIDs are divided into those that are selective (inhibiting only COX-2) and non-selective (inhibiting both COX-1 and COX-2). Selective drugs, called “coxibs”, have a less damaging effect on the gastric and duodenal mucosa and were initially used to prevent NSAID-associated damage to the digestive tract. However, it was later discovered that as gastrointestinal risks decrease when taking selective NSAIDs, the risk of fatal cardiovascular events increases [15,16,17]. Considering DIG, we should also note that the damaging effects of these drugs can be realized throughout the digestive tract and proceed with an awareness of the involvement of other organs and systems (liver, kidneys, etc.).

**Figure 1:**
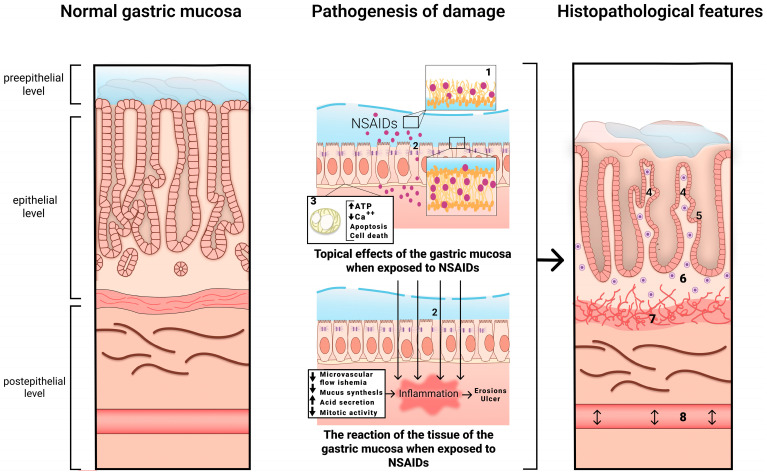
The mechanism of development of NSAID-associated gastropathy. 1—disrupted phospholipid monolayer, 2—damage to tight junction proteins, 3—uncoupled mitochondria, 4—pronounced regenerative changes in the epithelium (foveolar hyperplasia with hyperchromic cell nuclei, decreased mucus formation), 5—subnuclear vacuolated mucous cells, 6—mild diffuse mononuclear infiltration, 7—bundles of smooth muscle cells in the lamina propria, 8—edema with ectatic blood vessels.

The mechanism of development of NSAID-associated gastropathy is shown in Figure 1.

### 2.4. Clinical Manifestations

As a rule, most patients taking NSAIDs have no gastrointestinal symptoms. However, dyspeptic symptoms may occur in a significant number of patients, including epigastric pain (17–20%) and nausea (22%). In some cases, there might be symptoms such as heartburn, sour belching, constipation (19.3%) or diarrhea (9.2%) showing that other parts of the digestive tract are concerned [18].

A rare but clinically important feature of NSAID/aspirin-associated gastropathy is the development of complications, mainly gastrointestinal bleeding [19,20], with the risk of bleeding being greatest during the first 3 months of taking NSAIDs (OR 11.7; 6.5–21.0) and decreasing with the continued use, becoming minimal 1 week after the deprescribing (OR 3.2; 2.1–5.1) [21,22]. The absolute rate of peptic ulcer bleeding in patients taking these compounds has been reported to be 1% per year, but this rate may be increased substantially in patients with risk factors such as advanced age, a history of peptic ulcers and concomitant use of other drugs, such as anticoagulants, antiplatelet agents, corticosteroids and serotonin re-uptake inhibitors [4,21,22]. Bleeding ulcers can be indicated by the presence of hematemesis and/or melena, but some patients may report only general symptoms of blood loss such as a decrease in blood pressure, tachycardia, pallor of the skin, dizziness or anemia. Some patients with NSAID/aspirin-induced gastropathy may be asymptomatic.

It is important to note that damage to the digestive tract while taking NSAIDs/aspirin is not limited to the mucous membrane of the stomach and duodenum but can also affect the small and large intestines, as has been shown in a number of large, randomized clinical trials [23,24]. Most often, NSAID/aspirin-associated damage to the lower GI tract is accompanied by hidden blood loss and the development of chronic iron deficiency anemia, which aggravates the course of cardiovascular diseases and bronchopulmonary pathology and increases the risk of thromboembolic complications. NSAID/aspirin-associated enteropathy is accompanied, in addition to iron deficiency, by protein loss and hypoalbuminemia. A pathognomonic sign of damage to the small (rarely large) intestine, associated with the long-term use of NSAIDs, is the formation of circular, diaphragm-like strictures as a result of a chronic inflammatory process, which can cause intestinal obstruction [23,25,26].

### 2.5. Endoscopic Picture

A typical localization of erosive and ulcerative lesions is the antrum of the stomach, but all areas of the gastroduodenal tract can be affected. This condition is characterized by damage of a multifarious nature, which can be both acute and chronic. Signs of bleeding and subepithelial hemorrhages are often noted [3]. During the healing of an ulcer defect, as a rule, rough scars and deformities do not form [27].

### 2.6. Histological Examination

**Figure 2:**
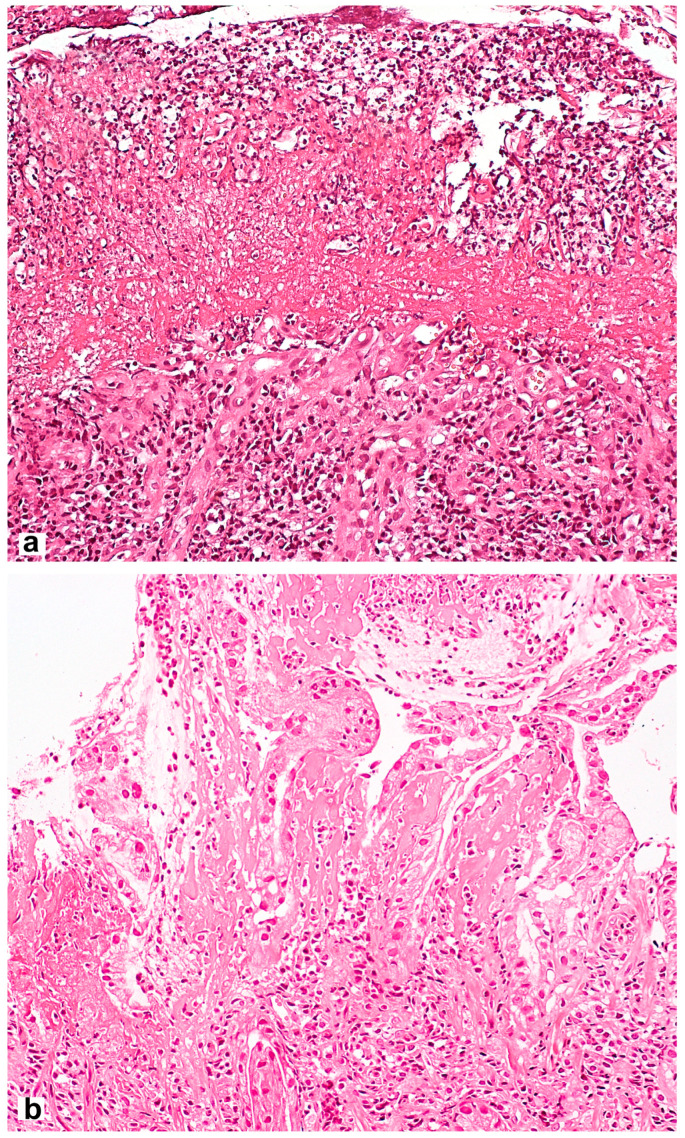
Differential diagnosis between NSAID-associated lesions and a defect caused by *Helicobacter pylori.* (**a**) Helicobacter pylori-associated gastric erosion. Inhomogeneous masses of fibrinoid necrosis with cell debris and granulocytes. (**b**) NSAID-associated gastric erosion. Homogeneous eosinophilic ischemic necrosis blending into the adjacent lamina propria. Hematoxylin and eosin stain ×200.

Microscopic signs comprise the picture of the so-called reactive gastropathy, which is not strictly specific to NSAIDs. There is a weak–diffuse, predominantly mononuclear inflammatory infiltration of the lamina propria, often revealing erosive and/or ulcerative defects, pronounced regenerative changes in the epithelium (foveolar hyperplasia with hyperchromic cell nuclei, decreased mucus formation), mucosal edema with vascular ectasia in the lamina propria and lamina propria expansion with fibromuscular proliferation. Subnuclear vacuolated mucous cells may be an additional criterion, which is associated with operated stomach syndrome. Interestingly, the nature of the necrotic masses at the bottom of the defect may be the starting point for the differential diagnosis between NSAID-associated lesions with a homogeneous eosinophilic zone of necrotic masses and a defect caused by *Helicobacter pylori* (*H. pylori*) [28] with the presence of necrotic masses that are loosely associated with the lamina propria, with immured fragments, necrotic cells and neutrophilic leukocytes (Figure 2). NSAID exposure, in very rare cases, is accompanied by the formation of diaphragms (diaphragm disease) in the stomach. This phenomenon is more typical of damage to the small and large intestines.

## 3. Anticoagulants

Anticoagulants are among the most commonly prescribed drugs worldwide. Over the past few decades, new second-generation oral anticoagulants (NOACs) that directly inhibit factor Xa (rivaroxaban, apixaban) or thrombin (dabigatran) have been introduced into a wide range of areas of clinical practice. They can be prescribed in fixed doses, without the need for laboratory monitoring, for the treatment and prevention of venous thrombosis and thromboembolism, including stroke, in non-valvular atrial fibrillation, while gastrointestinal bleeding is the “Achilles’ heel” affecting the application of this class of drugs [29].

### 3.1. Epidemiology

The possible negative risks of gastric mucosal damage in the establishment of anticoagulant therapy have been studied in numerous clinical trials, followed by evaluations in meta-analyses. At the same time, only the risk of upper digestive tract bleeding was studied as a phenomenon of interest without any assessment of its possible links with gastritis or gastropathy. In the latest review published in January 2023, which examined pharmacovigilance data registered with EudraVigilance [30], adverse reactions during treatment with anticoagulants were associated with bleeding in about half of the cases studied (n = 28,992/53,471). Of these bleeding events, >25% were associated with the gastrointestinal tract. The majority of patients with gastrointestinal bleeding were between the ages of 65 and 85 years old, with no clear differences between males and females. Gastric, ulcerative duodenal and rectal bleeding were the most common types of gastrointestinal bleeding, with fatal outcomes in 5.8%, 7.5% and 9.8% of cases with the use of rivaroxaban, apixaban and dabigatran, respectively.

An analysis of 16 RCTs showed that the greatest number of refusals to pursue further therapy occurred while taking dabigatran, while warfarin and factor Xa inhibitors slightly increased the incidence of adverse events involving the digestive tract [31]. A systematic review and meta-analysis published in 2019, including 43 RCTs with 183,752 patients, as well as data from real clinical practice (1,879,428 patients), showed no difference in the risk of major gastrointestinal bleeding between NOAC treatment and traditional treatment (warfarin or antiplatelet agents). However, in an indirect comparison of NOACs, rivaroxaban was associated with a 39% increased risk of gastrointestinal bleeding [32]. This meta-analysis found a non-significant association between dabigatran and an increased risk of major gastrointestinal bleeding (*p* = 0.95). In any case, there are multiple meta-analyses of either clinical trials or observational studies concerning the risk of gastrointestinal bleeding associated with NOACs and vitamin K inhibitors published in the last decade. All highlight an increased risk of gastrointestinal bleeding with the use of these agents with some differences between them, but these differences are of uncertain clinical significance.

### 3.2. Risk Factors

The major risk factors for gastrointestinal bleeding with NOACs are designated as follows [33]:

- A history of gastrointestinal bleeding;
- A history of gastric and/or duodenal ulcers;
- Gastroesophageal reflux disease, reflux esophagitis;
- Chronic H. pylori-associated gastritis;
- Other pathologies of the gastrointestinal tract: inflammatory bowel disease, diverticula, hemorrhoids and angiodysplasia;
- Neoplasms of the gastrointestinal tract in history;
- Age > 65 years;
- Concomitant use of NSAIDs (including LDA) or other drugs that affect blood coagulation or have a contact damaging effect on the gastrointestinal tract;
- Impaired renal function: glomerular filtration rate (GFR) < 50 mL/min;
- Use of high doses of NOACs (dabigatran 300 mg/day, edoxaban 60 mg/day).

However, some of the presented factors require further assessment and study. Thus, the consensus on H. pylori Maastricht VI, published in August 2022, states that there is no evidence to suggest that anticoagulants (coumarins, new oral anticoagulants and vitamin K antagonists) increase the risk of bleeding in patients with H. pylori infection (consensus level 91%, evidence level 1A) [34]. The potential impact of H. pylori infection on the risk of gastrointestinal bleeding is not well understood in patients taking anticoagulants. Further research is needed to understand the interaction between these two factors.

### 3.3. Mechanism of Gastric Damage

It is assumed that anticoagulants may increase the risk of bleeding from the gastrointestinal tract through several mechanisms or their combinations: (1) a systemic anticoagulant effect; (2) local anticoagulant effect; (3) local irritant effect; and (4) a local action of the drug not that is not associated with coagulation (for example, the inhibition of mucosal healing) [35,36,37].

The local and systemic anticoagulant effect is associated with the drug’s bioavailability. Thus, NOACs are characterized by low bioavailability (dabigatran 6%, apixaban 50%, rivaroxaban 60–80%); that is, a significant amount of the drug turns into an active anticoagulant during its passage through the gastrointestinal tract under the action of the intestinal esterases and, theoretically, can potentiate bleeding from vulnerable foci of pre-existing lesions (in combination with systemic action). In contrast, warfarin is absorbed at a level of more than 95%; thus, the increase in major gastrointestinal bleeding in patients taking warfarin probably reflects the systemic anticoagulant effect of the drug. The hypothesis that the tartaric acid in dabigatran may contribute to gastrointestinal bleeding due to a direct damaging effect seems unlikely.

### 3.4. Clinical Manifestations

The clinical manifestations of DIG resulting from the use of anticoagulants are highly controversial. On the one hand, anticoagulants, including aspirin, as well as NSAIDs are often taken simultaneously with anticoagulants. This situation highlights the need to study the risk of gastropathy development against the background of joint administration of anticoagulants with other potentially ulcerogenic drugs [38,39,40]. On the other hand, reviews and meta-analyses emphasize the importance of assessing hemorrhage as the major clinical complication [41,42,43]; thus, the clinical equivalent of gastropathy associated with anticoagulants is not clearly defined.

### 3.5. Endoscopic Picture

An upper endoscopy reveals changes ranging from erythema and petechiae to hemorrhagic gastropathy, Cameron’s lesions, erosion and stomach ulcers. The risks of peptic ulcers and bleeding from the upper gastrointestinal tract against the background of the use of NSAIDs and anticoagulants are presented in Table 1.

## 4. Cytostatics

Cytostatics comprise a group of antitumor drugs that are relatively heterogeneous in terms of their chemical structure and pharmacokinetic properties; therefore, data on the incidence of DIG and its development mechanisms, clinical course and endoscopic and morphological patterns are different for individual drugs in this group. In the literature, the greatest amount of data concerns DIG associated with the use of mycophenolate mofetil (MMF).

### 4.1. Epidemiology

Gastropathy and erosive and ulcerative lesions of the mucous membrane of the stomach and duodenum occur in 40–50% of patients taking MMF, while perforation and bleeding occur in 3–8% of cases, usually during the first 6 months after the start of MMF therapy [45,46].

### 4.2. Risk Factors

MMF intake is an independent risk factor for erosive and ulcerative lesions of the gastric mucosa, with an odds ratio of 1.83 (95% CI 1.02–3.29, *p* = 0.043), while the risk increases with combination therapy based on MMF with other cytostatics and/or glucocorticosteroids [47].

### 4.3. Mechanism of Gastric Damage

The mechanism of DIG associated with cytostatics is not clear. It is assumed that the damaging effect is due to the inhibition of cell renewal in the mucous membrane and the induction of cell death by drug metabolites [48]. The direct toxicity of acyl glucuronide, a by-product of MMF metabolism, to the gastric mucosa has been described [49]. It has also been established that cells exposed to MMF demonstrate an association with the dysfunction of the cellular cytoskeleton as a result of a decrease in the content of proteins contained within it: vinculin, actin and tubulin [50].

### 4.4. Clinical Manifestations

When taking MMF, 45–80% of patients experience decreased appetite, abdominal pain, nausea and vomiting [51,52]. In 40–50% of patients, gastrointestinal side effects are the main reason for dose adjustment, changes in the drug regimen or the discontinuation of immunosuppressive therapy [53].

### 4.5. Endoscopic Picture

Most often, in cases of erythema and edema of the gastric mucosa, multiple erosive lesions are detected. In some cases, the development of giant gastric and duodenal ulcers (more than 5 cm in size) is described [50,51].

### 4.6. Histological Examination

Characteristic morphological changes in the gastric mucosa against the background of cytostatic consumption include hyaline degeneration of cells in the submucosal and muscle layers and, in the case of large doses, cell vacuolization, necrotic changes and desquamation of the epithelium [54,55].

MMF-associated gastropathy is characterized by impaired mucosal architectonics with inflammatory infiltration and edema of the lamina propria of the gastric mucosa, the enlargement of the glands and increased apoptotic activity of the epithelium, less often resulting in changes resembling those observed in Crohn’s disease [52].

## 5. Immune Checkpoint Inhibitors

### 5.1. Epidemiology

Immune checkpoint inhibitors are a relatively new class of anticancer drugs; hence, data on the incidence of side effects resulting from their use are still limited. The first representative of this class, ipilimumab, was approved for the treatment of melanoma in 2011 [56]. It has been reported that 5% of patients taking immune checkpoint inhibitors experience mucosal damage limited to the stomach and duodenum [57].

### 5.2. Risk Factors

The risk of developing unwanted side effects affecting the digestive tract is determined primarily by the types of immune response checkpoint inhibitors used, among which are the inhibitors of cytotoxic T-lymphocyte-associated protein 4 (CTLA-4) and programmed cell death-1/programmed cell death ligand 1 (PD-1/PD-L1). Separate observational studies have shown that the incidence of gastric injury is higher for CTLA-4 than PD-1/PD-L1 and increases significantly with combination therapy [58,59,60]. The relative risk is also increased by the concomitant presence of *H. pylori* infection [61].

It is assumed that the risk of developing severe lesions of the stomach is increased in genetically predisposed individuals. Thus, a severe gastric lesion was described in a patient homozygous for the rs2241880 gene variant of the autophagy-related 16-like protein (ATG16L1), which is associated with Crohn’s disease [62].

### 5.3. Mechanism of Gastric Damage

The mechanism underlying the negative effects of inhibitors of immune response checkpoints on the gastric mucosa is not fully understood. It is assumed that during the use of this group of drugs, together with an increase in the antitumor activity of lymphocytes, an autoreactive immune response is activated against healthy tissues. A larger number of T- and B-lymphocytes are formed with the release of pro-inflammatory cytokines tropic to the gastric mucosa [63,64,65].

### 5.4. Clinical Manifestations

The complaints of patients taking immune response checkpoint inhibitors are nonspecific and correspond to dyspepsia syndrome. The main complaints include nausea, vomiting, abdominal pain and loss of appetite [62].

### 5.5. Endoscopic Picture

Endoscopic examination reveals erythematous and edematous gastric mucosa without obvious manifestations. The mucous membrane of the stomach is covered with a whitish fibrin-like film. When examining the body of the stomach with the applied magnification in narrow-spectrum imaging, the destruction of the glandular structure is visible. With air infusion, oozing hemorrhages are often noted, which indicates the friability of the mucous membrane [62].

### 5.6. Histological Examination

DIG associated with immune checkpoint inhibitor therapy exhibits two distinct patterns of damage [62]. The pattern observed in most cases has an outward resemblance to H. pylori-associated gastritis; however, unlike H. pylori-associated gastritis, it is diffuse in nature, captures not only the antrum but also the body, and is characterized by an increase in the number of interepithelial lymphocytes and a pronounced increase in the number of apoptotic bodies in the epithelium, often accompanied by ulceration. Another pattern is characterized by lymphoid infiltration, lymphocytes being the foci of cellular aggregates, often mixed with neutrophils and eosinophils resembling epithelioid granulomas, which may mimic granulomatous gastritis in infections, sarcoidosis or Crohn’s disease [62,66].

## 6. Glucocorticosteroids (GCSs)

### 6.1. Epidemiology

Data on the prevalence of gastropathy during GCS consumption are contradictory. Despite the generally accepted view that the use of corticosteroids increases the risk of developing peptic ulcers, large meta-analyses of randomized controlled trials have not shown a significant association between the use of corticosteroids and gastric ulcers [67]. Hence, according to the results of a meta-analysis published in 1994, combining the results of 93 placebo-controlled studies with the inclusion of 6602 patients, the incidence of peptic ulcers of the stomach while taking GCSs was 0.4%, which was comparable to that of the placebo group (0.3%) [68]. In general, the incidence of adverse events while taking GCSs, including gastropathy, dyspepsia and erosive and ulcerative lesions of the esophagus, stomach and duodenum, does not exceed 5%.

### 6.2. Risk Factors

The main risk factor for peptic ulcer bleeding in patients taking glucocorticosteroids is the concomitant use of NSAIDs/ASA. Recently, another study also reported an increased risk with the concomitant use of glucocorticosteroids and serotonin re-uptake inhibitors. Other risk factors for damage to the gastric mucosa while taking GCSs include older age (65 years and older), long-term use (a month or more) with a total intake of high doses (more than 1000 mg in regard to prednisone) and erosive and ulcerative lesions of the stomach and duodenum intestines, including those complicated by a history of gastrointestinal bleeding and *H. pylori* infection [67,69].

In the meta-analysis conducted by Narum S. et al., which combined data from 159 studies involving 33,253 patients, it was shown that an increased risk of gastrointestinal bleeding and ulcer perforation while taking corticosteroids is typical of only hospitalized patients, compared with outpatients receiving corticosteroids (40% versus 0.13% of cases) [70]. At the same time, additional risk factors are the severity of the course of the underlying disease, the presence of a severe comorbid pathology (diabetes mellitus, cancer) and the concomitant use of other drugs that damage the gastric mucosa [71]. In particular, epidemiological studies have shown that the relative risk of developing gastrointestinal complications is increased by 4–6 times in patients receiving corticosteroids together with NSAIDs [72,73].

### 6.3. Mechanism of Gastric Damage

GCSs inhibit the production of prostaglandin by regulating the activity of prostaglandin synthesis and the expression of type 2 cyclooxygenase [74]. It has been experimentally shown that under the action of GCSs, the production of mucins and the secretion of bicarbonates by the gastric mucosa decrease, which leads to a decrease in its resistance to aggressive factors [75] and the deterioration of the mechanisms of angiogenesis and epithelial repair [76]. In addition, corticosteroids increase gastric acid secretion and reduce peroxidase activity, with an increase in endogenous H_2_O_2_ levels being responsible for mucosal damage [77].

### 6.4. Clinical Manifestations

There are no specific clinical manifestations of this condition. The most common manifestations of dyspepsia syndrome are epigastric pain, nausea and a feeling of heaviness in the epigastrium. It is possible that the affected parents will be asymptomatic. The most severe manifestations include hemorrhagic gastropathy and erosive and ulcerative lesions of the stomach and/or duodenum, with the development of bleeding and posthemorrhagic iron deficiency anemia.

### 6.5. Endoscopic Picture

Single and multiple erosions with foci of hemorrhage and gastric or duodenal ulcers can be detected [78]. Ulcers are more often localized in the pyloric and prepyloric regions and are described as soft and pliable, with a weak fibrotic reaction [79]. Most of these lesions have been described in cases involving the concomitant use of other gastrotoxic drugs.

### 6.6. Histological Examination

The main possible manifestation of the action of GCSs on the gastric mucosa is the formation of erosive and ulcerative defects. This effect is most pronounced in combination therapy with NSAIDs [68,80].

A similar situation involving an increase in the effect of GCSs in combination with NSAIDs is reflected in the publications on gastric bleeding (upper gastrointestinal bleeding). This relationship has been demonstrated with particular clarity in patients with a history of previous bleeding [80]. In animal studies, the effect of GCSs on the hyperplasia of parietal and gastrin-producing G cells was shown, leading to a prolongation of the healing process of gastric ulcers (a delay in the healing of ulcers) [81].

## 7. Iron Drugs

The accumulation of iron in the gastric mucosa is known as gastric siderosis and was first described in the literature in the 1980s [82].

### 7.1. Epidemiology

The prevalence of iron-associated DIG is 0.7% in the adult population [83]. However, in 16% of patients taking oral iron preparations, iron granules are found in gastric biopsy specimens [84]. It is also worth noting that damage to the mucous membrane of the digestive tract during the consumption of iron preparations is not limited to the stomach and can occur in the duodenum [85].

### 7.2. Risk Factors

Studies have not been conducted on the search for risk factors for the development of gastritis induced by iron preparations. Based on the pathogenetic mechanisms underlying the effect of iron on the gastric mucosa, it can be assumed that concomitant diseases of the gastroduodenal zone may be predictors of more severe damage to the gastric mucosa while taking iron tablets.

### 7.3. Mechanism of Gastric Damage

Our knowledge of the pathogenesis of iron-associated DIG is limited. There are two hypotheses for the mechanism of damage. Firstly, iron can cause local effects in the gastric mucosa, mimicking a chemical burn. This is due to the fact that ferrous and ferric iron ions are catalysts for the formation of reactive oxygen species and highly toxic radicals, which can damage the components of the epithelial cells of the gastric mucosa [86,87,88]. In addition, the long-term intake of iron preparations may increase the concentration of free iron, which, in itself, is highly toxic in large doses and can lead to damage to various tissues [89].

### 7.4. Clinical Manifestations

The symptoms are nonspecific and may present as nausea, vomiting, abdominal pain or, in complicated cases, upper gastrointestinal bleeding [90].

### 7.5. Endoscopic Picture

Typical manifestations of iron-induced DIG are erythema, mucosal discoloration (brown, yellow or even cyanotic) and erosions [91].

### 7.6. Histological Examination

For the differential diagnosis of the side effects of iron tablets and other sideropenic conditions, a classification was proposed by Marginean et al., consisting of three models of iron deposition in the stomach. In the scheme of these authors, the most common pattern of gastric siderosis is the deposition of iron in the stroma and macrophages, mainly due to inflammation of the stomach, ulceration and previous bleeding and rarely due to iron supplementation. The second type is represented by extracellular iron deposition and is most often associated with the consumption of iron supplements. The third type is associated with iron overload and/or portal hypertension/cirrhosis, in which gastric cells are exposed to high concentrations of iron, possibly as a result of porto-caval shunting [86].

When staining biopsy specimens of the gastric mucosa with hematoxylin and eosin, characteristic features are chronic inflammation, the presence of superficial edema and a layer of brown granular pigment that covers the surface of the epithelium, spreads into the gastric pits, and may be present in macrophages localized in the lamina propria. By carrying out a histochemical reaction for iron (using Perls’ Prussian blue), it is possible to identify the substrate based on the appearance of a diffuse bluish color of the macrophage cytoplasm and the mucosal lamina propria [90].

The key features of DIG associated with different groups of drugs are presented in Table 2.

## 8. Proton Pump Inhibitors (PPIs)

Regarding DIG, it is necessary to make a few remarks about possible changes in the structure of the gastric mucosa in people who have undergone long-term PPI treatment. On the one hand, as part of the safety assessment of long-term PPI use, the risk of possible structural changes in the gastric mucosa is being actively studied and critically analyzed. On the other hand, these changes are difficult to interpret as either gastritis or gastropathy.

Undoubtedly, the most important changes have been described in relation to long-term acid suppression under the conditions of *H. pylori* colonization, namely a change in the topography of gastritis with an increase in the likelihood of atrophy of the gastric mucosa. In this regard, eradication is necessary for all patients with *H. pylori* infection undergoing long-term PPI therapy. However, these changes are probably related to the pro-carcinogenic potential of *H. pylori* rather than the independent effects of PPIs.

**Figure 3:**
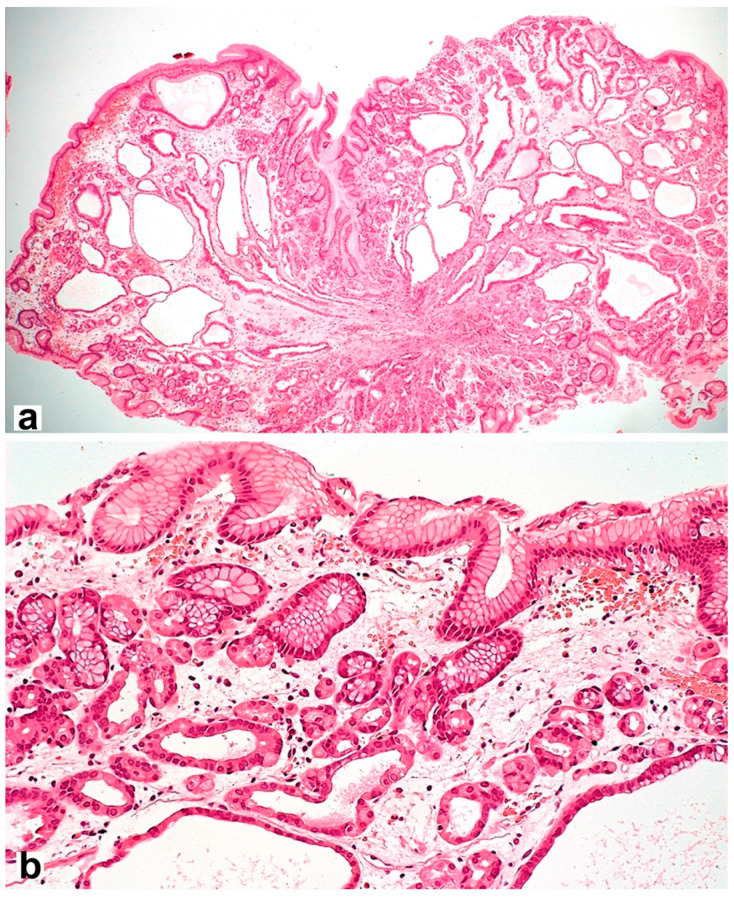
Fundic gland polyp with cystically dilated glands and hyperplastic parietal cells spreading up to the pits. Hematoxylin and eosin stain. (**a**) ×40, (**b**) ×200.

However, the long-term use of PPIs is associated with hyperplasia of enterochromaffin-like cells (ECL cells) and can provoke the formation of gastric fundus polyps [92] with specific morphological features (Figure 3). ECL cells play a key role in regulating gastric acid production through the release of histamine, which stimulates parietal cell acid secretion by binding to histamine-2 receptors. The risk of developing hyperplasia is likely to be influenced by both the duration and daily dose of PPIs, as well as the genetic factors of patients.

## 9. Conclusions

The expansion of opportunities for the management of both infectious and, above all, chronic non-communicable diseases is accompanied by an increase in the duration and number of drugs used by patients. The undoubted success of such treatment is accompanied by risks of drug damage to the digestive tract, including the stomach. For some of the drugs (e.g., NSAIDs/aspirin), sufficient experience has already been gained with regard to the possible manifestations, while for other, new groups of drugs (e.g., checkpoint inhibitors), we are only at the beginning of the journey towards the acquisition of scientific evidence. It is important to expand and understand not only the mechanism of damage and the risk factors but also the specific features of drug-induced gastrointestinal damage in order to prevent and recognize DIG in a timely manner.

## Figures and Tables

**Table 1:** Risks of peptic ulcers and bleeding from the upper gastrointestinal tract against the background of the use of NSAIDs and anticoagulants [44].

	Peptic Ulcers (OR)	Bleeding from the Upper GI Tract (OR)
NSAIDs	1.45	1.76
Coxibs	1.31	1.62
Aspirin (in small doses)	1.50	1.96
Antiplatelet agents (except aspirin)	1.53	1.82
Anticoagulants	1.62	2.38

**Table 2:** Characteristic features of DIG associated with various medications.

Medications	Clinical Manifestations	Endoscopic Signs	Histological Features
NSAIDs/Aspirin	▪ Dyspepsia syndrome (epigastric pain, nausea) ▪ Symptoms of involvement of other parts of the digestive tract: heartburn, sour belching, constipation, diarrhea ▪ A complicated course: signs of gastrointestinal bleeding, perforation and impaired patency of the small intestine	▪ Erythema ▪ Multiple erosions and ulcerative lesions with clearly defined edges, mainly in the antrum of the stomach ▪ Signs of bleeding, subepithelial hemorrhages ▪ Circular, diaphragm-like structures of the small (rarely large) intestine	▪ Weak–diffuse, mononuclear infiltration of the lamina propria of the mucosa ▪ Presence of erosive and/or ulcerative defect(s) ▪ Reactive gastropathy pattern ▪ Pronounced regenerative changes in the epithelium (foveolar hyperplasia with hyperchromic cell nuclei, decreased mucus formation) ▪ Edema with vascular ectasia in the lamina propria, detection of bundles of smooth muscle cells in the lamina propria
Anticoagulants	▪ Possible asymptomatic abdominal pain ▪ Nausea ▪ Diarrhea ▪ Hematemesis, hematochezia, manifestations of iron deficiency posthemorrhagic anemia in a complicated course (bleeding from erosive and ulcerative lesions)	▪ Erythema ▪ Petechial changes in the antrum mucosa ▪ Hemorrhagic gastropathy ▪ Cameron’s lesions ▪ Erosions and ulcers, mainly in the antrum of the stomach	▪ Reactive gastropathy pattern (see above) ▪ Presence of erosive and/or ulcerative defect(s)
Glucocorticosteroids	▪ Possibly asymptomatic ▪ Dyspepsia syndrome (epigastric pain, nausea, feeling of heaviness in the epigastrium) ▪ Hematemesis, hematochezia, signs of iron deficiency anemia in a complicated course (bleeding)	▪ Single and multiple erosions with foci of hemorrhage ▪ Ulcerative lesions of the stomach, more often in the pyloric and prepyloric regions or duodenum	▪ Presence of erosive and/or ulcerative defect(s)
Mycophenolate mofetil (cytostatics)	▪ Loss of appetite ▪ Abdominal pain ▪ Nausea ▪ Vomiting	▪ Spotted erythema ▪ Mucosal edema ▪ Multiple erosive lesions ▪ Giant gastric and duodenal ulcers (rare)	▪ Hyaline degeneration of cells in the submucosal and muscular layers ▪ Vacuolization of cells, necrosis and desquamation of the epithelium (when taking large doses) ▪ Inflammatory infiltration and edema of the lamina propria ▪ Expansion of damaged glands and increased apoptotic activity of the epithelium ▪ Histological changes resembling those of Crohn’s disease (rare)
Immune checkpoint inhibitors	▪ Abdominal pain ▪ Nausea ▪ Vomiting ▪ Loss of appetite	▪ Erythema ▪ Mucosal edema without manifestations ▪ Whitish, fibrin-like film on the surface of the mucous membrane ▪ When viewed in the NBI mode, the destruction of the glandular structure is visualized ▪ Oozing hemorrhages are often noted	▪ A pattern that has an outward resemblance to H. pylori-associated gastritis (H. pylori-like changes); however, unlike H. pylori-associated gastritis, it has a diffuse character, captures not only the antrum but also the body, and is characterized by the presence of intraepithelial lymphocytosis and pronounced apoptosis, often accompanied by ulceration ▪ A pattern resembling granulomatous gastritis secondary to infections, sarcoidosis or Crohn’s disease, with characteristic lymphoid proliferation and the presence of histiocytes in focal lesions, often mixed with neutrophils and eosinophils resembling epithelioid granulomas
Iron preparations	▪ Nausea ▪ Vomiting ▪ Abdominal pain, signs of bleeding in a complicated course	▪ Erythema ▪ Change in the color of the mucous membrane (brown, yellow or even cyanotic) ▪ Erosive damage to the mucous membrane	▪ Superficial mucosal edema and determination of a layer of brown granular pigment that covers the surface of the epithelium, extends into the gastric pits, and may be present in macrophages localized in the lamina propria (when stained with hematoxylin and eosin) ▪ Multifocal positive reaction to iron deposition, which is characterized by a diffuse bluish tint of the cytoplasm of macrophages and the mucosal lamina propria (Perls’ stain)

## Data Availability

Not applicable.
